# Exploring self-rated health among adolescents: a think-aloud study

**DOI:** 10.1186/s12889-016-2837-z

**Published:** 2016-02-16

**Authors:** Junia Joffer, Lars Jerdén, Ann Öhman, Renée Flacking

**Affiliations:** Department of Public Health and Clinical Medicine, Epidemiology and Global Health, Umeå University, Umeå, SE-901 87 Sweden; Center for Clinical Research Dalarna-Uppsala University, Nissers väg 3, Falun, SE-791 82 Sweden; School of Education, Health and Social Studies, Dalarna University, Falun, SE-791 88 Sweden; Umeå Centre for Gender Studies, Umeå University, Umeå, SE-901 87 Sweden

**Keywords:** Self-rated health, Subjective health, Health assessment, Feel, Adolescence, Qualitative, Think-aloud interview, Cognitive interviewing, Sweden

## Abstract

**Background:**

Despite extensive use of self-rated health questions in youth studies, little is known about what such questions capture among adolescents. Hence, the aim of this study was to explore how adolescents interpret and reason when answering a question about self-rated health.

**Methods:**

A qualitative study using think-aloud interviews explored the question, “How do you feel most of the time?”, using five response options (“Very good”, “Rather good”, “Neither good, nor bad”, “Rather bad”, and “Very bad”). The study involved 58 adolescents (29 boys and 29 girls) in lower secondary school (7th grade) and upper secondary school (12th grade) in Sweden.

**Results:**

Respondents’ interpretations of the question about how they felt included social, mental, and physical aspects. Gender differences were found primarily in that girls emphasized stressors, while age differences were reflected mainly in the older respondents’ inclusion of a wider variety of influences on their assessments. The five response options all demonstrated differences in self-rated health, and the respondents’ understanding of the middle option, “Neither good, nor bad”, varied widely. In the answering of potential sensitive survey questions, rationales for providing honest or biased answers were described.

**Conclusions:**

The use of a self-rated health question including the word ‘feel’ captured a holistic view of health among adolescents. Differences amongst response options should be acknowledged when analyzing self-rated health questions. If anonymity is not feasible when answering questions on self-rated health, a high level of privacy is recommended to increase the likelihood of reliability.

## Background

There is widespread agreement in the literature that self-rated health measured as a single-item general health question is an important health indicator and a strong predictor for future outcomes such as morbidity and mortality [1–4]. However, there is no unanimous phrasing of a self-rated health question, and response options also vary. Several different concepts are in use, such as self-rated health, subjective health, self-perceived health, and self-assessed health. For example, in the SF-36 instrument, respondents are asked to evaluate their “health in general” using a five-point scale from *excellent* to *poor* [5]; in the instrument EQ-5D respondents rate their “health state today” using a 100-point visual analogue scale from *worst imaginable* to *best imaginable* [6]; and the World Health Organization (WHO) study, Health Behavior in School-aged Children (HBSC) asks adolescents to describe their health (“Would you say your health is…”) based on a four-grade ordinal scale from *excellent* to *poor* [7]. Despite the different wording of self-rated health questions, they all seem to reliably predict future health outcomes [1, 8].

During adolescence, self-rated health decreases with age, but boys report good health to a greater extent than girls [7]. In Sweden, 47 % of 11-year-old boys and 42 % of 11-year-old girls report “very good” health, while among 15-year-olds these figures decline to 37 % among boys and 22 % among girls [9]. Both short- and long-term quantitative studies of adolescents’ self-rated health suggest the concept is multidimensional. A Canadian youth study found that self-rated health was predicted by an overall sense of functioning, based on a combination of personal, socio-environmental, behavioral, psychological, and physical health factors [10]. In the adolescent population of the Norwegian Nord-Trøndelag Health Study (Young-HUNT), associations with medical, psychological, social, and lifestyle factors were found [11].

Although self-rated health is known to predict a range of health outcomes, less is known about how respondents interpret the questions being asked. Almost two decades ago, Idler and Benyamini concluded that the field of self-rated health research must address specific groups using qualitative approaches [2]. Furthermore, Jylhä has argued that a better understanding of the cognitive processes of health assessments in general is needed [12]. Qualitative studies of adults have shown that self-rated health questions comprise aspects of health behaviors, physical functioning, health problems, and emotions [13–16]. Despite extensive research on self-rated health, there are very few qualitative studies that have explored the question of self-rated health from a youth perspective. Studies using cognitive interviewing techniques have suggested that children as young as 8 years old are able to adequately report on different aspects of their health [17–19]. Yet, little has been documented about their actual interpretations of self-rated health questions. A U.S. study showed that young people (14–24 years old) were more likely to refer to health behaviors, while older people were more likely to mention health problems, suggesting that people in different age groups use different frames of reference [20]. In contrast to a small child’s concrete way of thinking, an adolescent thinks in more abstract ways. This cognitive development occurs during adolescence, and enables more complex reflection [21]. As cognitive functions are rapidly developing during adolescence, it is important to include participants from the entire adolescent age range.

Given the reliable evidence showing that self-rated health is an important health indicator, there is much to be gained from a better understanding of what a self-rated health question captures from a youth perspective. This insight could help us interpret the results of many quantitative youth studies that use such questions. Hence, the aim of this study was to explore how adolescents interpret and reason when answering a question on self-rated health.

## Methods

### Study design

A qualitative, inductive, research design with the interviewing technique ‘think-aloud’ was used. Think-aloud interviews seek to understand the strategies that respondents use to answer questions, and to detect potential sources of response errors within the questions [22]. With this technique respondents are asked to say everything out loud from the moment they see the question until they give an answer, and subsequent probes (follow-up questions) are often used [22]. Respondents can be instructed to report their thinking concurrently (i.e., while answering the question), or retrospectively (i.e., immediately after answering the question or finishing the questionnaire). In the present study, a combination of the two methods was used, a technique that has been suggested for producing optimal data quality [23].

### The self-rated health question

In the present study, the young age of the study population guided the choice of question. In contrast to the use of the word ‘health’, as described in the various questions in the background, the present study focused on the word ‘feel’ (‘må’). In Sweden, the word ‘feel’ is often used when talking about health in everyday language, and sometimes used in questionnaires for adolescents, e.g. the national mapping of children and adolescents’ mental health [24] and in local school-health surveys in Sweden. The reliability of the question has been assessed in a test-retest [25], showing a kappa value of 0.54. The complete wording of the self-rated question in the present study was: “A person may feel good sometimes and bad sometimes. How do you feel most of the time?” (In Swedish: “Man kan må bra ibland och dåligt ibland. Hur mår du för det mesta?”). Five response options followed the question: “Very good”, “Rather good”, “Neither good, nor bad”, “Rather bad”, and “Very bad.” In order to get an optimal translation of the question for the publishing of the study, the question was translated from Swedish to English by academics who spoke English fluently and whose mother tongue was Swedish. Alternative wordings were then discussed with native English-speaking academics, who were also asked to elaborate on how they believed adolescents would perceive these terms. Finally, in an effort to obtain the most appropriate translation, adolescents in England were asked to describe how they perceived the wording of the question.

### Setting and sample

The study was conducted in a town in Sweden (population 56,000). The town is representative of the country with respect to educational levels and adolescents’ self-rated health [24, 26]. Participants included adolescents in lower secondary school (12–13 years old, 7th grade) and upper secondary school (17–18 years old, 12th grade). In an attempt to include participants from a variety of socioeconomic backgrounds, students were recruited from two lower secondary schools and one upper secondary school with academic, vocational, and introductory school programs. To assess gender differences and similarities, both boys and girls were invited to participate.

The study was approved by the headmasters of the schools. School nurses and class teachers of 11 classes were informed about the study before students were contacted. Classes were selected in consultation with the headmasters of the schools. Students were approached for recruitment during class hours, and provided with both oral and written information about the study. Students agreed to participate in the study and for data to be published by signing a consent form: students in upper secondary school had the option of consenting immediately upon receiving the information, or returning their consent form later; students in lower secondary school who agreed to participate signed the consent form, but were also required to obtain informed consent from their parents.

A total of 58 interviews were conducted, characteristics of the participants are described in Table 1.

**Table 1:** Characteristics of the study participants

	Lower secondary, *n* = 23	Upper secondary, *n* = 35
Gender		
Boys	10	19
Girls	13	16
Country of birth		
Born in Sweden	22	30
Born outside of Europe	1	5
Self-rated health		
Very good	9	14
Rather good	13	19
Neither good, nor bad	1	1
Rather bad	-	1
Very bad	-	-

### Data collection

To test the think-aloud technique and the research protocol, pilot interviews were conducted (not included in the analysis). All of the interviews took place in quiet rooms in the different schools. The interviews lasted between 20 and 90 min, a total of 31.5 h. Interviews with older respondents tended to last longer. All interviews were performed in Swedish, although English were sometimes used to help clarify statements in interviews with respondents not having Swedish as their native language. The interviews were recorded, and then transcribed verbatim. After each interview, the participants received a movie ticket.

The interviews comprised two different topics: self-rated health (the subject of this article) and subjective social status. For each interview, the section about subjective social status was initiated only after a participant had completed the whole section about self-rated health. To practice the think-aloud technique, a set of neutral questions were asked such as “How do you consider the weather today?”, and “What do you think about dogs?” Respondents were asked to imagine answering the question alone, like a questionnaire is usually answered (i.e., not in the presence of an interviewer). When the respondent felt comfortable and understood how the interview would be performed, a sheet of paper was presented, stating the self-rated health question and corresponding response options.

The interview guide comprised four parts. First, in the concurrent think-aloud phase, the respondent was asked to say out loud everything s/he thought about when answering the question. If the respondent answered the question without thinking out loud, s/he was encouraged to retrospectively think aloud, by using the probe: “What were you thinking about when answering the question?” Second, in the retrospective phase, the following probes were used: “Did you say everything that you thought about? Did you get a direct feeling of which option you would choose? Were you sure about your answer?” Third, semi-structured interview questions were used to determine, for example, why they chose one alternative instead of another, and how honestly they felt they answered potentially sensitive survey questions. Finally, to explore possible differences in how the respondents perceived the words ‘feel’ and ‘health’, respondents were asked to elaborate on what they would have answered if they had been asked about their ‘health’.

### Data analysis

Interviews were analyzed using qualitative content analysis [27]. The first (JJ) and last (RF) authors performed separate coding of all the data. Interviews were divided into four groups, and color-coded: younger girls, older girls, younger boys, and older boys. Both the division and the color-coding helped illuminate potential differences and similarities between boys and girls of different ages. Interview transcripts were read several times. Meaning units were identified, labeled with codes and sorted into content areas. Based on this initial analysis, categories were developed on both a manifest level (i.e., clearly reasoned and obvious information) and a latent level (i.e., underlying meanings of the text). While the first (JJ) and last (RF) authors conducted most of this analysis, all of the authors discussed the content of the interviews, were involved in the analytical phase, and contributed to the interpretation of the text.

### Ethical considerations

Ethical considerations were taken into account during every phase of the study. The participants were informed about their right to end the interview at any time, and were free to ask questions both before and after the interview. After the interviews, the participants were asked how they felt about being interviewed and were also informed about the option of visiting the school nurse/counselor. The study was approved by the Regional Research Ethics Committee at Uppsala University (dnr 2011-110). The study adheres to the RATS guidelines for reporting qualitative studies.

## Results

### The interpretation and essence of the question

#### The concept of ‘feel’

The age of the respondents influenced the way they answered the question. That is, while older respondents often made complex interpretations and detailed reasoning (e.g., moving back and forth in time), younger respondents used a more uncomplicated and direct way to describe how they felt. Younger respondents more often related their self-rated health to recent, everyday, events such as “I spilled dressing on my sister’s pizza”, or “I lost the key to the house.” Older respondents made attempts to define the concept of ‘feel’; as one girl said to herself, “What do you mean by feel…physically or mentally or what.” (Girl, 12th grade)

For most respondents, the question captured a holistic view that included social, mental, and physical aspects of ‘feel’. Participants reflected on social and mental aspects, and described them in detail, but used shorter statements to describe physical aspects. The self-rated health in both boys and girls of different ages were clearly influenced by social aspects. Participants emphasized relationships with friends, parents, and siblings and older respondents also highlighted the importance of their relationship with a significant other. Moreover, respondents often discussed the social aspects of ‘feel’ in relation to mental aspects. One boy (12th grade, answered “Rather good”) exemplified this by describing his situation at home:

My family have not felt so good lately, both financially and relation wise. Like when you come home, you can almost always expect that there will be nagging. It is just like ahhhh. Sometimes we even start to shout at each other.

Social and mental aspects were also intertwined throughout the adolescents’ views on peer relations, which were described as both stressful and empowering. This was especially crucial for younger girls who expressed deep concerns about not fitting into their peer group; affirmation from peers was important to feel good, and being included in their peer group was a sign of such affirmation. In the school context, participants viewed teachers as significant for the creation of a positive atmosphere within peer groups. However, participants also often described teachers as having failed at this task. One of the more significant school-related influences on how participants felt was stress caused by homework. While all groups evidenced this, older girls reported to a greater extent that a heavy workload in school affected them particularly. One girl described her situation: “I take it [homework] with me on the weekends… sometimes I even forget to eat… It’s too much, school takes up my life.” (Girl, 12th grade, answered “Neither good, nor bad”) Participants also experienced stress in relation to societal norms, for example future education, job opportunities, and gender issues. One girl (12th grade) described the pressure she felt in choosing a proper education, and adapting herself to social and gendered expectations:

The whole society as it looks… a lot of what you think is wrong and strange… if you see a woman in town, it is assumed that she should enjoy wearing make-up, she’s supposed to clean, she should do this and that. When you meet a guy, you should assume that he thinks it is really fun to build houses and drink beer and do macho stuff. We have expectations of things and I think it can be hard sometimes.

Physical aspects such as health behaviors, injuries, and illness also influenced how respondents felt. They referred to participation in sports as a fun activity, but also as something that made the body feel better. Younger boys frequently mentioned spare-time activities, and emphasized the importance of getting enough sleep to cope with long days. Those participants who had recently suffered from an injury or illness were most likely to discuss the impact that injuries and illnesses had on how they felt.

#### Contrasting ‘feel’ and ‘health’

When participants were asked if they would have given the same response if the question had included the word ‘health’ instead of ‘feel’, they overwhelmingly related ‘health’ to behaviors such as physical activity, food habits, and tobacco use. They also regarded illness, e.g. having a cold or being sick in some other way, as a factor that influences one’s ‘health’. One boy in 12th grade who reported that he felt “Very good” when the question included the word ‘feel’, reflected on how his answer might have differed if the question had included the word ‘health’ instead:

No, then I would have responded “Rather good.” I watch what I eat and I work out. But then I use *snus* [Swedish moist snuff]. And I’m like… I drink maybe a bit too much during the weekends. I know it is not possible to do so all my life. But my health is still very good, I think at least. I eat a lot of vegetables and get everything I need. I exercise and get fresh air… Health for me, it’s more about how I take care of my body and everything like that… purely chemical. Just how the body feels, but not how I feel as a person.

When viewing the concepts of ‘feel’ and ‘health’ in contrast to one another, participants referred to ‘feel’ primarily as a mental concept, which was valued more than physical aspects. However, they also described the concepts as interrelated:

If I had to describe it in short, I guess I would say that health is more physical and how you feel is more mental. That is to say, health is how you feel in your body. And how you feel, it’s more… I mean they are intertwined. If you don’t feel good mentally, then your health gets worse and then it will be a bad circle. And the same goes the other way, if you are less healthy you feel worse. If I had to choose, I would rather have slightly worse health but feel very good than the other way around. (Boy, 7th grade, answered “Rather good”)

### Strategies for determining a response

Four strategies were identified for determining subjective health: they made judgments by *stating*, *comparing*, *weighing*, and *summarizing*. Some respondents simply *stated* that they felt good; that is, they seemed to have an instant feeling that they felt good. Such a direct expression was more common among the younger respondents. Others made more complex evaluations, for example, by *comparing* how they felt at present with past experiences. They made these comparisons in relation to time, not to peers. One girl in 7th grade exemplified this by comparing her current situation with that of her situation in 6th grade:

In my former class, it was very much so that the girls were supposed to be girly, and the guys were supposed to be macho. And then I didn’t fit in…So then I was bullied a bit, so now I feel like almost everything is better than back then.

Participants also formed judgments by *weighing* positive and negative experiences. One girl in 12th grade reflected upon the way in which positive home and school experiences became clouded by the heavy workload in school, and thus influenced her judgment:

In reality it is very good. I go to a very good school. I have very nice classmates. I have good teachers, only good teachers. And then I also have a very good relationship with my mother. I have very good relationships with my roommates and so on. But it is also very stressful in school. The schedule is packed. We have a lot of homework. And it feels like there is no time. What should I say I feel… how does one do if it feels pretty good, but also pretty bad… I’ll circle this one [“Neither good, nor bad”].

Finally, participants made *summations*, by adding together the aspects they deemed most important: they drew from either positive or negative aspects (e.g., “a good school, nice friends, helpful parents”, or “poor sleep, too much homework, absent parents”). One girl summarized using percentages, stating that she felt good 90 to 95 percent of the time.

### Choosing a response

#### Finding an appropriate response option

For some participants, the response option “Very good” represented a positive state with occasional negative feelings. Others regarded the option as a constant state, and used the phrase “all of the time”, rather than reiterating the phrase “most of the time”, as it was written in the questionnaire: “You can’t feel good all the time, then you live in an illusion.” (Boy, 12th grade, answered “Rather good”) Hence, for some, this response option described an extreme value, a utopia, or a state in which everything is perfect.

The response option “Rather good” was an attractive choice for many respondents: those who seemed to feel good but regarded “Very good” as an unattainable state; and those who referenced many negative elements in their life but seemed to perceive lower response options as too negative. Others, deemed “Rather good” a normal but mediocre state, while some respondents perceived “rather” as negative, and opted for the more appealing option of “Good”: “I want to choose ‘Good’, ‘rather’ sounds a bit negative.” (Boy, 12th grade)

Respondents considered the response option “Neither good, nor bad” as partially negative: “That is a little more negative, I would say… if you do not know whether you feel good or bad.” (Boy, 7th grade) However, they also described it as a statistical mean with terms such as “in the middle”, “normal”, “neutral”, and “50/50.” By contrast, some participants regarded it as a passive state, a response that indicated not caring. One girl in 12th grade described it as being “in limbo”, while another 12th-grade girl expressed a wish for a different wording and suggested “Sometimes good, sometimes bad.”

Although only one respondent chose the option “Rather bad”, all of the respondents reflected out loud on how they perceived it: as a very negative response, associated with mental illness and bullying. Some regarded it as bad, but not excessively so: “That you feel bad most of the time. But you might sometimes still feel quite ok. In general however, you feel bad.” (Boy, 12th grade)

Respondents perceived “Very bad”, in a similar way as “Very good”, as a type of extreme value and in some cases as a constant state. They suggested when and why that option might be applicable, e.g. when living with a lifelong disability, feeling apathetic toward life, or experiencing a recent death in the family.

#### Attitudes toward providing an honest answer

Our findings showed that the final aspect influencing the participants’ choice of response options was their attitude toward providing an honest answer. We observed both honest and biased responses when answering the question. Respondents who provided an honest answer claimed that being honest was not an issue, and that it did not matter whether the survey was anonymous and/or conducted in private: “I don’t understand why I wouldn’t do it [give an honest answer]. If you are doing a survey, it must be filled out properly. You cannot make things up and lie.” (Girl, 12th grade, answered “Very good”) Other participants described why they gave a biased answer. First, to prevent ‘others’ from seeing their answers. Participants explained that they were more likely to be honest, and to choose a less flattering response option (when relevant) if the questionnaire was anonymous. Furthermore, respondents were concerned about the risk of being offered help from an adult if there was the slightest chance that they could be identified. Respondents also said that providing honest answers depended on their sense of confidentiality; that is, the option of answering the questionnaire in private, without the risk of peers or teachers seeing their answers. For example, one girl in 12th grade explained that she would probably give more honest answers if she answered the questionnaire at home. The second reason participants gave for providing a biased answer was the fear of confirming their negative feelings to themselves. Admitting on paper that one feels badly seemed to make such feelings too definite; ticking a better (i.e., more positive) option seemed to be one way of dealing with such a situation:

No… I would not like to admit it in writing, so that it sticks. Because even if you look down on yourself… you do not want to admit you are at the bottom, it’s like to confirming that it’s like that. (Girl, 7th grade, answered “Rather good”)

## Discussion

In this study, we found that the use of the word ‘feel’ in a self-rated health question captured a holistic health construct in adolescents that included social, mental, and physical aspects. We identified gender and age differences that may be potentially important for our understanding of how adolescents interpret such questions. Two of these differences were: girls’ emphasis on experiences of stressors (i.e., gender difference), and older respondents’ inclusion of a wider variety of influences on their assessments. Furthermore, our results indicate that the response options represent differences in subjective health and that participants’ level of honesty in providing potentially sensitive survey answers varies. We discuss these findings below.

### A holistic health construct

In this study of adolescents’ self-rated health, our findings suggest that differences in question wording should be acknowledged. Previous research show that different wording of such questions seems to represent equivalent assessments [1, 8]. The present study used different wording, in contrast with prior studies: ‘feel’ instead of ‘health’. We chose to explore ‘feel’, as we found this word more appropriate for our young study population, and for the Swedish context. In Sweden, ‘feel’ is used as a synonym for ‘health’ [16] and represents a word that is used in everyday language. Both ‘feel’ and ‘health’ are used in self-rated health questions in Sweden [9, 24]. Our findings show that ‘feel’ captures a holistic health construct in adolescents, an interpretation that aligns with the definition of ‘health’ made by the WHO: “…a state of complete physical, mental, and social well-being…” [28]. When contrasting the concepts of ‘feel’ and ‘health’, we found that participants primarily referred to ‘feel’ as a mental concept, while they focused on health behaviors and physical aspects when referring to ‘health.’ One possible explanation for this close association of ‘health’ with health behaviors is that the Swedish school subject, “Sports and Health”, requires students to practice sports and study topics that mainly relate to health behaviors and lifestyle factors. Another possible explanation is the influence of the media, who often conflate ‘health’ with fitness, exercise, and healthy eating. It is also possible that participants’ responses were influenced by the particular sequencing of different wording during the interviews. For example, we explored ‘health’ near the end of the interview, when the respondents had already been talking about ‘feel’. Hence, the respondents might have felt obligated to give a different response than the one they gave to ‘feel’. Nonetheless, most respondents provided spontaneous answers and seemed certain about their responses.

### Gender and age differences

Think-aloud interviews seek to understand the strategies that respondents use to answer a survey question [29]. When assessing the validity of a survey question, differences in respondents’ reasoning about and interpretations of the question should be discussed. If differences are too extensive, it could indicate weak validity, and lead to difficulties in interpreting the results. In the present study, we found gender and age differences. However, when addressing gender differences, there is a risk of reproducing such differences by assuming differences when there are none (i.e., gender bias) [30]. Similarly, genuine differences can be overlooked when assuming sameness between boys and girls [31]. We were careful to account for these sameness/difference biases in our analysis, as explicated in gender studies [32].

In this study, we found that the main gender difference was girls’ focus on stressors in relation to self-rated health. Participants associated stress with achievements such as finishing homework, excelling in school, gaining future education, and finding jobs. This observation is in line with Wiklund’s [33], who attributes similar stressors to modernity, gender orders and youth. Theoretically, this understanding of gender adheres to a constructionist view of gender, in which gender orders create different forms of femininities and masculinities [34]. Another significant stressor that we found in the present study was younger girls’ fear of being excluded from their peer group. Johansson and colleagues [35] underscore the importance of relational factors, and regard them as one of the most important aspects associated with feeling good among adolescents in Sweden. This was particularly the case among younger respondents (13-year-olds), who regarded friends as the most important factor. In the present study, respondents described peer relations as a struggle, as they felt that they had to adapt themselves to fit into their peer group. Flacking and colleagues [36] observed a similar phenomenon when they found that avoiding rejection and striving for acceptance were key elements in adolescent girls’ sense of well-being. We do not think that these gender differences weaken the validity of our question; we have shown, rather, that these aspects merely seem to influence girls’ assessments of subjective health more than they influence boys’ assessments.

The main difference we observed between age groups was the relative complexity of the assessments older respondents made when answering the questions. Similar findings were described in a Swedish interview study about adolescents’ understandings of “mental health”, in which the younger respondents seemed to have more difficulty describing the concept [35]. This finding also agrees with Piaget’s explanation of the development during adolescence of an abstract way of thinking [21]. Furthermore, increasing age implies increasing responsibilities, and thus often more aspects of life to consider as one approaches adulthood. For example, the number of stressors regarding future education and job opportunities arguably begin to expand. Hypothetically, the older respondents’ inclusion of a wide variety of aspects influencing their assessments, and the girls’ emphasis and reflections on stressors, might enhance our understanding about why self-rated health deteriorates during adolescence and why more girls than boys rate their health as poor in later adolescence.

### The response options

Our results indicate that each of the five response options in this study represent differences in subjective health; these differences should be considered when analyzing self-rated health in quantitative studies. Some differences are inevitably lost when variables are dichotomized. Our participants’ understandings of the response alternative “Neither good, nor bad” varied in this study. Some regarded it as normal and “in the middle”, some as a negative alternative, and others as a passive state. Similar conclusions were made by Schytt and colleagues [16]. Our respondents’ unwillingness to pick this option may have resulted in an overly positive estimation of the alternative “Rather good.” However, we believe that phrases such as “Both good and bad”, and “Sometimes good, sometimes bad”, might also be problematic, as they tend to be too general (e.g., who does not feel both good and bad at some point?). The WHO [35] suggests “Fair” as another response alternative. “Fair” is also used in the SF-36 instrument, although as the fourth out of five options [5]. It would be beneficial to further investigate adolescents’ understandings of different response options. Perneger and colleagues [37] argue that a visual analogue scale with descriptions at the extremities of the scale (like the EQ-5D), may provide more consistent data across socioeconomic groups. However, the WHO does not recommend using scales, as they may have different meanings in different cultures [38].

### Honest and biased answers

When answering a survey question, respondents in general are reluctant to give answers that diverge from the norm (i.e., responses that are socially undesirable). Sudman and colleagues [23] suggest, therefore, that researchers address this issue at the end of an interview when validating survey questions through interviews (e.g., by asking about possible difficulties with the question). Additionally, we found in the present study that participants were more likely to give (more) honest answers in an anonymous survey (i.e., no name stated; and conducted in a private environment). This observation is in line with the WHO’s emphasis on the importance of providing a non-threatening and confidential environment that allows children to be honest when answering surveys [38]. One of our participants suggested that inviting respondents to answer a questionnaire at home would result in more honest answers. However, this benefit must be evaluated in view of the potential for lower response rates typical of postal surveys. In the present study, some respondents described why they would provide a biased answer; that they did not want to admit to others or to themselves that they felt badly. Previous studies have found that boys who feel badly tend to not choose the lowest (i.e., most negative) option when answering questions about how they feel [35]. In our study, both boys and girls discussed this matter. We suggest that resistance towards providing honest answers when participating in surveys could produce an under-representation of adolescents who are feeling badly.

### Methodological considerations

The findings from this study support previous research on different processes that are involved when answering a survey question. For example, Sudman and colleagues [23] described a theoretical four-task model. The first task of this model involved the respondents’ interpretation of the question (e.g., understanding of specific words or the meaning of the entire question); the second task covered the respondents’ retrieval of information from their memory; the third task concerned how a respondent forms a judgment; and the fourth task dealt with how a respondent translates their judgment into a response (e.g., editing their answer). All steps of this well-recognized theoretical framework were, in large, identified in the present study. Interviews with short concurrent think-aloud sections, including only a sentence or two, were the exceptions. Despite an inductive way of performing the analysis, i.e. not performing the analysis through the theoretical framework, the four steps were in large confirmed. This validates Sudman and colleagues’ theory in that it also seems applicable to the adolescent population.

One aspect of a think-aloud technique that researchers should discuss is the relatively richer descriptions given by people with greater verbal facility [23]. We observed this phenomenon in our study as older respondents were able to share more complex assessments than younger respondents. As the interview progressed into a retrospective think-aloud most respondents were able to give richer descriptions. We attributed this improvement to our prompts for clarification of their interpretations. The combination of concurrent and retrospective think-aloud techniques, with probes, was central to our acquisition of rich data.

To assess the trustworthiness of a study, findings should be evaluated in relation to the procedures used to generate data [39]. One strength of this qualitative study was the relatively large number of participants. Including both boys and girls of varying ages enabled us to explore a sameness/difference approach based on gender and age. Our sample included adolescents from different ethnic groups, from different upper secondary school programs, and from various classes in different lower secondary schools. We believe these factors improved the credibility of the study, and the transferability of the results. The two separate initial codings of all interview transcripts, and the interdisciplinary contribution of co-authors to the discussion of the results, further enhanced the credibility of the study. We asked all the respondents the same research questions which strengthened the dependability of the study [27]. The use of the word ‘feel’ may have different meanings in different cultures, and thus affect transferability of the results. We suggest, therefore, that researchers aiming to capture holistic health constructs for adolescents in other languages, make an effort to identify wordings that captures the same aspects that “feel” does in Sweden. Furthermore, adolescents’ understandings of the word ‘health’ in other cultures may be associated with other factors than those we explored in this study.

One limitation of this study was the small number of adolescents who claimed low self-rated health. Efforts to identify respondents with poorer self-rated health were made by recruiting through the introductory programs in upper secondary school, and through the school counselors in lower secondary school. Rather few Swedish adolescents report low self-rated health (e.g. 6–7 % in a recent study using the same question [40]), making the recruitment of adolescents with lower self-rated health more difficult. However, we believe that a sufficient numbers of respondents were able to reflect about the more negative response options (as they had had previous experiences of ill-health), to achieve credibility.

## Conclusions

Our findings showed that the use of the word ‘feel’ in a self-rated health question captures a holistic view of health in adolescents. Neither gender-related differences (e.g., girls’ reflections about stressors) nor age-related differences (e.g., older respondents’ wider inclusion of aspects influencing their assessments) changed the overall holistic view. The differences amongst respondents’ interpretations of the concepts ‘feel’ and ‘health’ suggest that question wording should be carefully considered when investigating adolescents’ self-rated health, taking into account which aspects one wishes to capture. Our findings also indicate that each of the five response options we used encompass differences in subjective interpretations of health; future analyses should acknowledge this. Finally, we recommend that precautions be taken to ensure a high level of privacy for survey participants, to enhance the validity of sensitive surveys. Recommendations for the use of the question are described in Table 2.

**Table 2:** Recommendations for the use of a single-item self-rated health question in adolescents

Recommendations
1. Carefully consider the focus of the study before choosing the wording of a question. The word “feel” seems to capture a holistic construct, whilst “health” may imply emphasis on physical aspects.
2. Acknowledge differences between response options when analyzing a self-rated health question. If dichotomization is necessary, thoughtful consideration of the grouping of variables is encouraged.
3. To enhance the reliability of a self-rated health question, appropriate precautions should be taken to ensure privacy.

### Availability of data and materials

As data consist of qualitative interviews with sensitive personal information, data will not be shared.
